# Carrier Localization Effects in InGaN/GaN Multiple-Quantum-Wells LED Nanowires: Luminescence Quantum Efficiency Improvement and “Negative” Thermal Activation Energy

**DOI:** 10.1038/srep34545

**Published:** 2016-09-30

**Authors:** Wei Bao, Zhicheng Su, Changcheng Zheng, Jiqiang Ning, Shijie Xu

**Affiliations:** 1 Department of Physics, Shenzhen Institute of Research and Innovation (SIRI), and HKU-CAS Joint Laboratory on New Materials, The University of Hong Kong, Pokfulam Road, Hong Kong, China; 2 Mathematics and Physics Centre, Department of Mathematical Sciences, Xi’an Jiaotong-Liverpool University, Suzhou 215123, China; 3 Suzhou Institute of Nano-tech and Nano-bionics, Chinese Academy of Sciences, Suzhou 215123, China

## Abstract

Two-dimensional InGaN/GaN multiple-quantum-wells (MQW) LED structure was nanotextured into quasi-one-dimensional nanowires (NWs) with different average diameters with a combination approach of Ni nanoislands as mask + dry etching. Such nanotexturing bring out several appealing effects including deeper localization of carriers and significant improvement in quantum efficiency (e.g., from 4.76% of the planar MQW structure to 12.5% of the 160 nm MQW NWs) of light emission in the whole interested temperature range from 4 K to 300 K. With the aid of localized-state ensemble (LSE) luminescence model, the photoluminescence spectra of the samples are quantitatively interpreted in the entire temperature range. In terms of distinctive temperature dependence of photoluminescence from these samples, a concept of “negative” thermal activation energy is tentatively proposed for the MQW NWs samples. These findings could lead to a deeper insight into the physical nature of localization and luminescence mechanism of excitons in InGaN/GaN nanowires.

It has been known for a long time that carrier localization is a ubiquitous phenomenon in real solid-state materials due to lattice disorder, impurities, and various imperfections including defects and deep centers, and thereby has a profound impact on both the electrical and optical properties of materials^1^. For example, the localized energy states caused by In composition fluctuations and In-rich nanostructures in InGaN alloy working as the active layer of efficient blue light-emitting diodes (LEDs) are closely related to the high efficiency of this kind of LEDs which is the key element in solid-state lighting technique revolution^2^,^3^. Usually, InGaN/GaN planar multiple-quantum-wells (MQW) structure acts as the active layer (light emitting layer) in the InGaN-based blue LEDs. To date, luminescence efficiency of InGaN/GaN MQW LEDs is still insufficient to support such a lighting technique revolution^4^. Moreover, efficiency droop problem in the operation of the blue LEDs under large injection current is a severe hinder^5^,^6^. Very recently, InGaN/GaN MQW nanorods or nanowires (NWs) have been proposed and explored as the active layer in order to achieve higher luminescence efficiency^7^,^8^,^9^,^10^,^11^,^12^. Some encouraging results, e.g., enhanced light extraction efficiency due to creation of the interspaceal regions between the wires and improved luminescence internal quantum efficiency due to suppression of the quantum confined Stark effect (QCSE) by partial relaxation of the lattice mismatch strain, have been achieved in these available studies. Even reduced exciton-phonon (e.g., longitudinal optical mode) coupling has been claimed by Renwick *et al*. in the post-growth nanotextured InGaN/GaN MQW nanorods^13^.

On the other hand, nanotexturing InGaN/GaN MQW planar structure into nanowires may bring out some other fascinating properties and phenomena such as new surface electronic states, new surface vibrational modes etc^14^,^15^. Nevertheless, an in-depth investigation on localization, luminescence efficiency and mechanism of carriers in InGaN/GaN MQW nanowires is still lacking and highly desirable in terms of both fundamental and technological interests. In the present article this is done. We first examine the variable-temperature photoluminescence (PL) of InGaN/GaN MQW LED structure before and after the nanotexturing, and then make theoretical quantitative analysis to the experimental data with localized-state ensemble (LSE) luminescence model. Good agreement between theory and experiment enables us have a deeper insight into the physical nature of luminescence mechanisms in the InGaN/GaN MQW nanowires. We also propose a concept of “negative” thermal activation energy for describing anomalous temperature behaviors of luminescence of the InGaN/GaN MQW nanowires. These state-of-the-art findings and arguments could be commonly valid for very different materials with carrier localization.

From the top-view ((a) and (c)) and the tilted-view ((b) and (e)) SEM images of the two NWs samples in Fig. 1, it can be seen that the NWs are about 160 nm ((a) and (b)) and 100 nm ((c) and (d)) in diameter on average, respectively, and both are about 700 nm in height. Nanowire sheet densities of the 160 nm and 100 nm NWs samples are estimated to be 1.1 × 10^7^ and 2.6 × 10^7^ cm^−2^, respectively. Figure 2 shows the micro-Raman spectra of the three samples at room temperature. Compared with the as-grown planar MQW sample, the two NWs samples exhibit an additional Raman scattering peak located at the lower energy side of the longitudinal optical (LO) phonon (A_1_-LO, 736.23 cm^−1^ ≈ 91.3 meV) mode. Moreover, frequency dependence of this additional Raman mode on the nanowire size such as diameter distinctively follows the theoretical prediction on the surface optical phonon mode localized on the surface of nanoscale crystals^14^,^15^,^16^,^17^. The appearance of the surface optical phonon mode indicates that the post-growth nanotexturing can alter significantly the optical properties of InGaN/GaN planar MQW structure. As addressed earlier, localization and luminescence mechanism of excitons in the nanotextured InGaN/GaN MQW structures are the central issue of the present study. Following discussion and arguments will concentrate on this issue.

Figure 3 illustrates the 3.6 K PL spectra of the as-grown planar MQW sample (solid line), 100 nm (solid line + solid circles) and 160 nm (solid line + open circles) NWs samples. The inset figure shows the close up view (semi-logarithmic scale) of the PL spectra near the band-edge transitions of GaN. After the nanotexturing, both significant effects, namely noticeable blue-shift in PL peak position and large enhancement in intensity, are observed. The blue-shift in PL peak position has been referred to as the largely reduced QCSE effect due to the obvious reduction of the original strong piezoelectric field by partial release of the lattice mismatch strain in the quantum wells^7^,^8^,^9^,^10^,^13^. As for the significant enhancement in PL intensity, there are two possible reasons: (1) increase of the internal quantum efficiency (IQE) of luminescence due to the increased oscillator strength caused by the band profile flattening; and (2) enhancement of the external light extraction efficiency due to the open volumes among the nanowires^10^. In addition to the peak blue-shift and emission intensity increase, amazing transition of the PL lineshape from the broad multiple peaks before the nanotexturing to the almost single peak after the nanotexturing could be a more interesting phenomenon, which has not yet addressed in the literature. In our opinion, it may suggest a remarkable change in the localization and luminescence mechanism of carriers after the nanotexturing. As shown below, by examining temperature-dependent PL spectra of these samples, we may obtain a deeper insight into the carrier localization and luminescence mechanism in the samples, in particular in the two NWs samples.

To save space, we just depict the 3.6 K (Fig. 3) and 300 K PL spectra (Fig. 4) of the as-grown planar MQW sample (solid line) and the two NWs samples (solid lines + symbols). Compared with the 3.6 K PL spectra, the 300 K PL spectra of all the three samples broaden significantly. We now extract all the three chief spectral parameters, namely peak position (spectral center), integrated intensity and linewidth, from the measured variable-temperature PL spectra of the three samples. The obtained data are depicted in various solid symbols in Figs 5, 6 and 7 for the three samples. For the planar sample, we use a single Gaussian lineshape function to do a best fit to the measured PL spectra for getting the chief spectral parameters. As seen in Figs 6 and 7, S-shaped temperature dependence of the PL peak positions is observed for the two NWs samples, which is a typical temperature behavior of collective luminescence of localized excitons^18^,^19^. Taking into account generation, thermal activation (e.g., non-radiative recombination, radiative recombination, and re-capturing of excitons, as well as assuming a Gaussian-type density of states for localized-state ensemble, a generalized luminescence model for localized-state ensemble (LSE) was developed by Li and Xu *et al*.^18^,^19^,^20^. According to this model, temperature-dependent PL peak positions can be written as


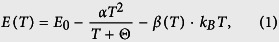


where *β*(*T*) is a temperature-dependent dimensionless parameter which can be obtained by numerically solving the following equation:


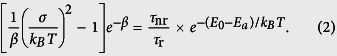


Here *α* is Varshni’s parameter, Θ Debye temperature, *τ*_n*r*_ time constant of thermal activation (e.g., non-radiative recombination), *τ*_*r*_ time constant of radiative recombination, *E*_0_ and *σ* energetic center and width of the Gaussian-type density of states for localized-state ensemble distribution, respectively, and *k*_*B*_ the Boltzmann constant. From the model itself and its application practice, Δ*E* = *E*_0_ − *E*_*a*_ is found to act as a more important role than both parameters themselves in determining temperature behavior of luminescence. Depending on materials and experimental conditions, Δ*E* can take positive (+) or negative (−) sign^20^. For the InGaN/GaN material systems investigated in the present work, Δ*E* usually takes a positive sign^18^,^19^. Actually, if luminescence quantum efficiency *η* is defined as 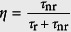, Eq. (2) can be re-written as


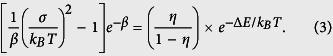


By using Eqs (1) and (3), we may get several chief parameters characterizing luminescence of localized excitons via making a best fit to the temperature dependence of PL peak position. Parameters adopted in the fitting are tabulated in Table 1. A unified Debye temperature Θ = 630 K is taken in the fitting for all the three samples. As mentioned earlier, noticeable blue-shift in peak position and large increase in emission intensity are the two significant effects observed after the nanotexturing. The former is evidenced by the increase of parameter *E*_0_. For example, it increases from 2.7850 eV of the planar MQW structure to 2.8065 eV of the 160 nm NWs sample, further to 2.8472 eV of the 100 nm QWs sample. This phenomenon has been well documented and interpreted as the QCSE weakening effect largely due to the strain relaxation induced by nanotexturing^7^,^8^,^9^,^10^,^13^. However, for the luminescence intensity increase after the nanotexturing, its origin has not yet well understood. In the present study, the luminescence quantum efficiency is obtained for the three samples. From Table 1, the luminescence quantum efficiency of the two NWs samples is much higher than that of the planar MQW sample. In particular, the luminescence efficiency of the 160 nm NWs sample is about 2.6 times stronger than that of the planar sample. Such result is very encouraging because enhancement of the luminescence quantum efficiency is our central objective. But, when the nanowires’ average diameter is reduced to about 100 nm, the luminescence quantum efficiency drops, i.e., from 12.5% of the 160 nm NWs to 7.69% of 100 nm NWs. This efficiency decrease could be attributed to the enhanced surface state effect leading to nonradiative recombination in small size nanowires.

It has been known for a long time that the S-shaped temperature dependence of the PL peak position is a characteristic behavior of localized carriers in semiconductors. By using the LSE luminescence model, the S-shaped temperature dependence of the PL peak positions of both the 160 nm and 100 nm NWs samples can be well reproduced, as shown in Figs 6 and 7. As argued earlier, Δ*E* = *E*_0_ − *E*_*a*_ rather than *E*_*a*_ itself plays a more significant role in determining abnormal temperature dependence of luminescence of localized carriers. For the as-grown planar MQW LED structure, its Δ*E* = 11.2 meV is much smaller than parameter *σ* = 19.80 meV and the luminescence linewidth. This is the main reason why the PL peak of the planar sample doesn’t show obvious S-shaped temperature dependence. Instead of, the peak positions show a “chair” shaped temperature dependence due to large parameter *σ* (characterizing the distribution width of localized states), as seen in Fig. 5. After the planar structure was nanotextured into 160 nm nanowires, however, Δ*E* significantly increases to 14.3 meV. For the resulting 100 nm NWs, Δ*E* further increases to 27.2 meV. It should be noted that in general cases, Δ*E* may take negative sign^20^. That implies that *E*_*a*_ is usually above *E*_0_ in which localized carriers are thermally activated to states (or sites in real space) with higher energies, as schematically shown as dashed horizontal line in Fig. 8. In such cases, Δ*E* takes a negative value, but is usually assigned as “positive” thermal activation energy. However, in the InGaN/GaN system, especially in their nanowires, Δ*E* takes a positive value. We thus argue that the thermal quenching of luminescence in InGaN/GaN nanowires may be characterized by “negative” thermal activation energy in terms of their abnormal temperature dependence of luminescence peak and positive Δ*E*. In Fig. 8, we schematically illustrate such cases, e.g., *E*_*a*_ below *E*_0_, then Δ*E* = *E*_0_ − *E*_*a*_ takes a positive value, but defined as a “negative” thermal activation energy. In the figure, the wavy arrows stand for the thermal activation processes, while the straight downward arrow represents luminescence transition. The bottom curve represents a luminescence spectrum of localized-state ensemble. For the studied NWs samples, as argued earlier, strongly reduced QCSE effect takes place due to the strain relaxation and hence piezoelectric electric field reduction. As a result, *E*_0_ could rise and the absolute value of Δ*E* might increase. Indeed, our experimental results demonstrate this tendency. In order to further verify the occurrence of largely reduced QCSE effect in the NWs samples, we conducted the variable-temperature PL measurements on both the NWs samples under the different excitation intensities. Shown in Fig. 9 are the peak positions (symbols) of measured PL spectra of the 160 nm and 100 nm NWs samples against temperature for three different excitation powers. As expected, the PL peak shifts toward higher energy direction as the excitation intensity increases, especially at low-medium temperatures. Again, we employ Eqs (1) and (2) to make a fit to the experimental data, and good agreement between theory and experiment is achieved. The solid lines represent the fitting curves.

In addition to the peak position, full width at half maximum height (FWHM) or simply linewidth is another key parameter which also carries important information on localization mechanism of carriers^20^. Reduction in linewidth with temperature in a temperature range is another PL signature of carrier localization principally due to the thermal redistribution of localized excitons among different localized states^18^,^20^. According to the LSE model, the thermal distribution of localized excitons is jointly determined by Gaussian-type density of states and a newly-derived distribution function. In mathematic form, the thermal distribution of localized excitons can be described by *n*(*E, T*) = *ρ*(*E*) · *f*(*E, T*). Here *ρ*(*E*) = *ρ*_0_ exp[−(*E* − *E*_0_)/2*σ*^2^] is a standard Gaussian function and 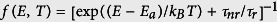 is a newly derived distribution function for localized carriers^20^. It has been proven that *n*(*E, T*) essentially represents the spectral profile of localized-state ensemble luminescence^20^. The FWHM Γ_*c*_(*T*) of *n*(*E, T*) is thus obtained by numerically solving 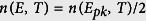. Besides, phonon scattering induced broadening shall be taken into account. By doing so, we can write down the expression for the actual linewidth of localized-state ensemble luminescence:


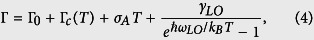


where Γ_0_ is a temperature-independent constant accounting for scattering by unavoidable impurity and imperfection^21^,^22^, *σ*_*A*_*T* represents the broadening term induced by acoustic phonon scattering which is linearly dependent on temperature^21^, and the last term on the right side of Eq. (4) stems from optical phonon scattering. In polar semiconductors like GaN, the dominant scattering mechanism arises from LO phonon mode^21^,^22^. *γ*_*LO*_ represents exciton-LO phonon coupling strength, while 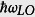 is the characteristic energy of LO phonon. By using Eq. (4), we have made a fit to the experimental linewidth data. Parameters adopted in the fitting are tabulated in Table 2. It can be seen from Figs 5, 6 and 7 that good agreement between theory and experiment is achieved in the entire interested temperature range for all the three samples. Compared with the as-grown planar MQW sample, both the NWs samples likely show weaker exciton-LO-phonon coupling, e.g., 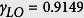 eV for the 100 nm NWs sample, while 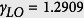 eV for the planar MQW sample. Our results seem to be consistent with Renwick *et al*.’s experiment^13^. In another previous work done by Chen *et al*., significant enhancement of electron-LO-phonon coupling was observed in highly strained InGaN/GaN quantum well structures in terms of the relative intensity between the first-order LO phonon replica and its principal emission line^23^. They attribute such enhancement to the interface features such as interface optical phonon mode and the high residual strain. In our case, noticeable release of the residual lattice-mismatch strain and new surface optical phonon mode located at the lower frequency side of A_1_-LO mode (See, Fig. 2) are observed in both the NWs samples. Therefore, weaker exciton-LO-phonon coupling is observed in the InGaN/GaN nanowires.

At last, we want to make some argument on the third key parameter of a luminescence spectrum, namely integrated intensity. Following the LSE model, the integrated intensity of localized-state ensemble luminescence can be described by^20^


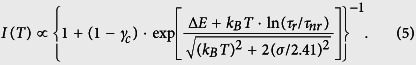


By substituting parameters tabulated in Table 1 into Eq. (5), we compute the temperature dependence of the measured luminescence intensity and depict the results as solid curves in Figs 5, 6 and 7. Reasonably good agreement between theory and experiment is achieved for all the three samples. It should be pointed out that the re-capture coefficient *γ*_*c*_ plays an important role in the temperature dependence of luminescence intensity for localized-state ensemble in addition to *E*_0_ − *E*_*a*_ and 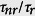
or quantum efficiency *η*.

In conclusion, by measuring PL spectra of the 160 nm and 100 nm InGaN/GaN MQW nanowires, as well as the as-grown planar MQW sample under the same conditions, we demonstrate that luminescence intensity is greatly enhanced in the entire interested temperature range after the nanotexturing to the planar MQW LED structure. With the aid of the LSE model, we have acquired several key physical quantities including internal quantum efficiency, distribution width of localized states, and an energy parameter for the localized excitons in the planar sample and nanotextured nanowires. Indeed, great enhancement of the internal quantum efficiency is revealed to be responsible for the observed luminescence intensity improvement after the nanotexturing. “Negative” thermal activation energy is tentatively proposed to interpret the abnormal temperature dependence of luminescence in InGaN/GaN nanowires. As a final remarking, we shall point out that localization and luminescence mechanism in InGaN/GaN heterostructures including QW and nanowires represent an extremely complicated and challenging issue, which still has a distance from full understanding.

## Experimental Section

The starting sample used in this study was a typical MQW LED structure consisting of layers in the order from top to bottom: a 0.2 *μm* p-type GaN:Mg top layer, then 5 periods of In_0.2_Ga_0.8_N/GaN MQWs with barrier’s (well’s) thickness of 30 nm (3 nm), and a 3.0 *μm* n-type GaN:Si buffer bottom layer, which was grown on c-plane sapphire with metalorganic chemical vapor deposition. The nanowires were prepared with the “top-down” dry etching process using Ni self-assembled nanoislands as the etching mask. Other preparation details of the NWs can be referred to a publication^24^.

A JEOL (JSM-7001F) SEM microscopy was employed to characterize the dimensions of the nanopillars. Micro-Raman scattering spectra were measured on an integrated WITec Alpha scanning confocal micro-Raman system with the back-scattering geometric configuration^14^. In the Raman scattering measurements, the 514.5 nm line of an argon ion gas laser was used as the excitation source. Variable-temperature PL spectral measurements were carried out on a home-made PL system using a Kimmon 325 nm He-Cd laser as the excitation light source. The maximum output power of the He-Cd laser was 38 mW. In the PL measurements, the samples were mounted on the cold figure of a Janis closed cycle cryostat proving a varying temperature range of 3.6–300 K. A detailed description of the PL setup can be referred to a previous publication^25^.

## Additional Information

**How to cite this article**: Bao, W. *et al*. Carrier Localization Effects in InGaN/GaN Multiple-Quantum-Wells LED Nanowires: Luminescence Quantum Efficiency Improvement and “Negative” Thermal Activation Energy. *Sci. Rep.* **6**, 34545; doi: 10.1038/srep34545 (2016).

## Figures and Tables

**Figure 1:**
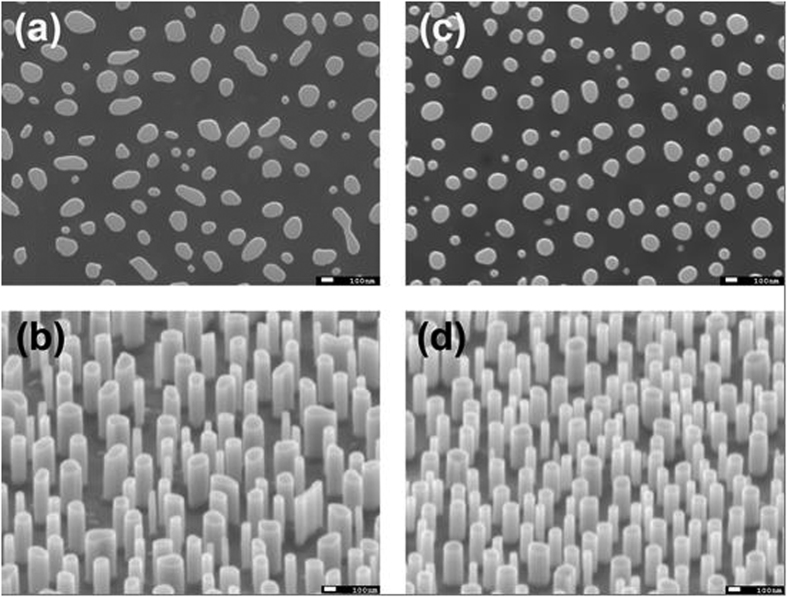
Top-view and tilted-view SEM images of the two nanowires samples. (**a**,**b**) for the 160 nm NWs, while (**c,d**) for the 100 nm NWs.

**Figure 2:**
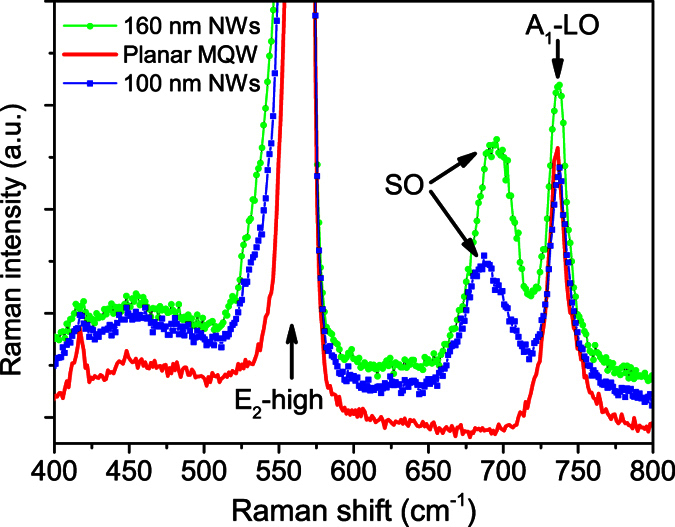
Micro-Raman spectra of the three samples measured at room temperature and under the back-scattering geometric configuration. New surface optical phonon mode (denoted as SO) can be clearly observed for the NWs sample.

**Figure 3:**
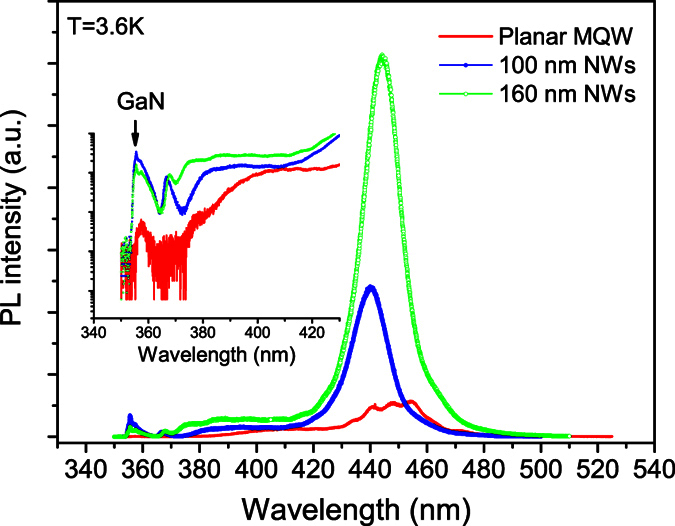
3.6 K PL spectra of the three samples measured under the same conditions. The inset shows close-up spectra near the band-edge emission of GaN. Compared with the planar MQW sample, both NWs samples show significantly improved emission intensity at low temperatures, especially the 160 nm NWs sample.

**Figure 4:**
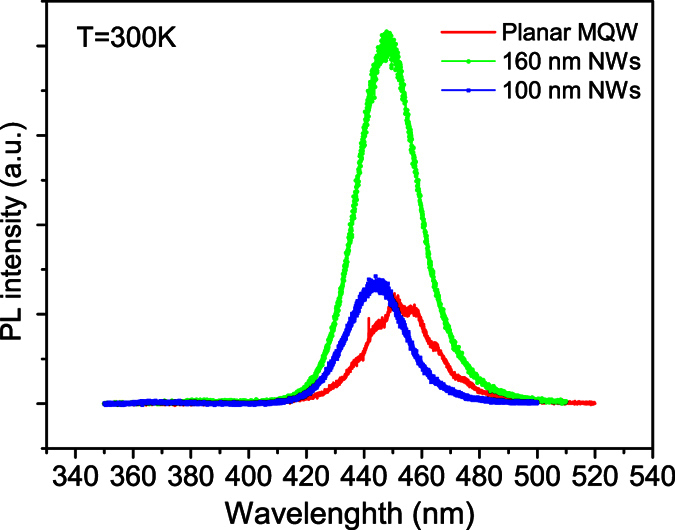
Room-temperature PL spectra of the three samples. Again, the NWs samples exhibit improved emission intensity at room temperature. In particular, the 160 nm NWs sample shows a substantial improvement in PL intensity.

**Figure 5:**
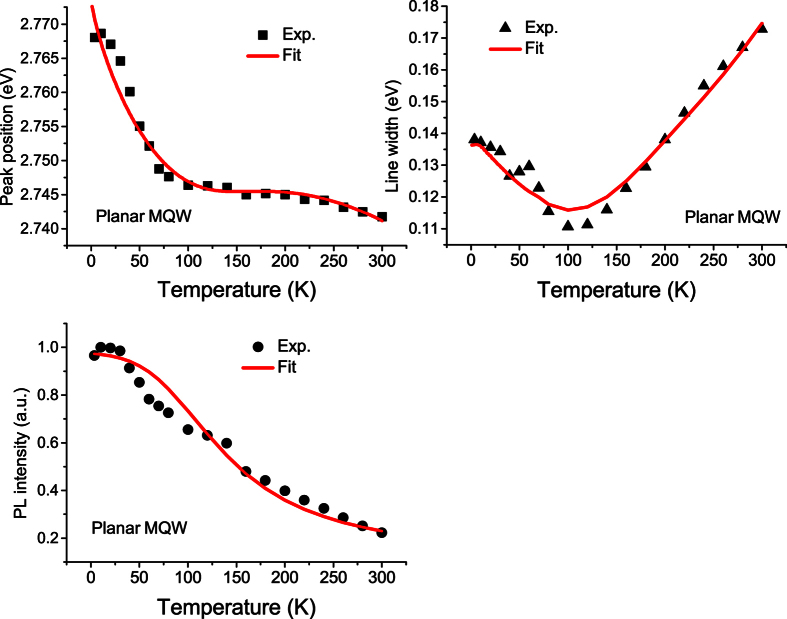
Extracted peak positions, FWHM and integrated intensities of the planar MQW sample at different temperatures. The solid lines are the fitting curves with LSE model.

**Figure 6:**
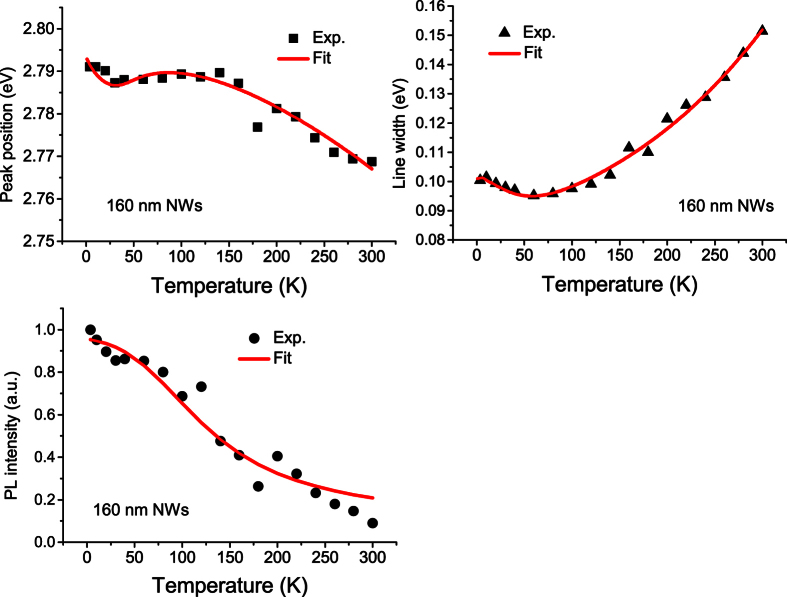
Extracted peak positions, FWHM and integrated intensities of the 160 nm NWs sample at different temperatures. The solid lines are the fitting curves with LSE model.

**Figure 7:**
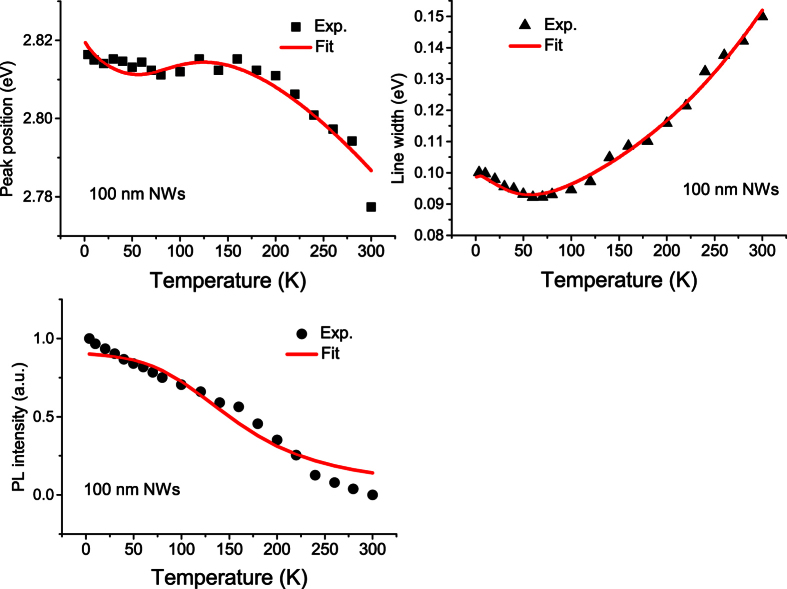
Extracted peak positions, FWHM and integrated intensities of the 100 nm NWs sample at different temperatures. The solid lines are the fitting curves with LSE model.

**Figure 8:**
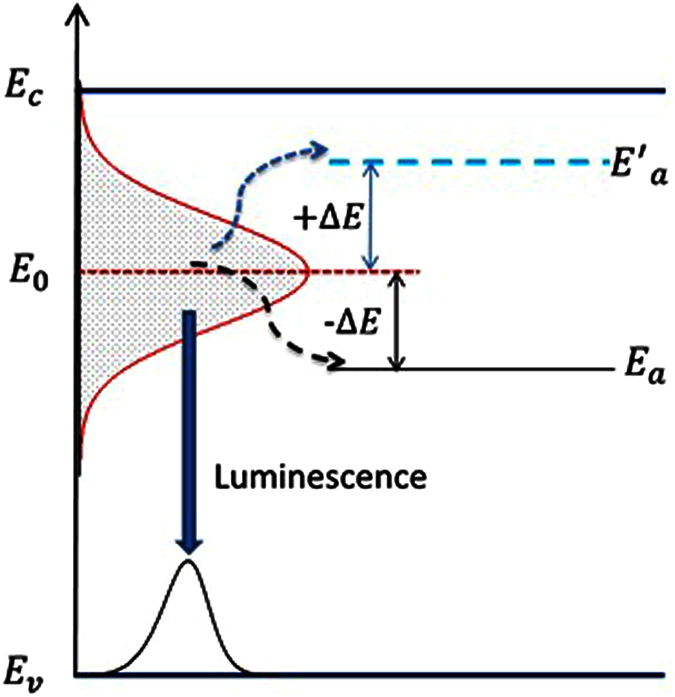
A physical picture showing the luminescence mechanism of localized-state ensemble. Several key energy parameters are denoted, especially +*ΔE* and −*ΔE* which represent positive and negative thermal activation energy, respectively, corresponding to the normal thermal quenching and abnormal thermal quenching of luminescence.

**Figure 9:**
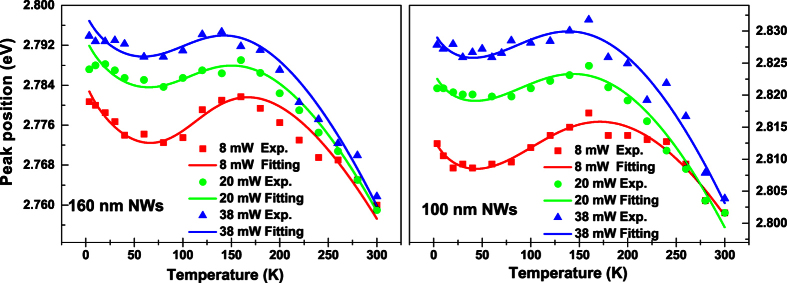
Peak positions (symbols) of the measured PL spectra vs. temperature for the 160 nm NWs and 100 nm NWs samples for three different excitation powers. The solid lines are the fitting curves with LSE model.

**Table 1:** Parameters for the three samples.

Sample	*σ* (meV)	*E*_0_ (eV)	Δ*E*(meV)	*η*	*γ*_*c*_	*α*(meV/K)
160 nm NWs	11.10	2.8065	14.3	12.5%	0.21	0.451
100 nm NWs	16.30	2.8472	27.2	7.69%	0.36	0.643
Planar MQW	19.80	2.7850	11.2	4.76%	0.34	0.245

**Table 2:** Parameters for the fitting to FWHM data of the three samples.

Sample	*σ*_*A*_(meV/K)	*γ*_*LO*_ (eV)	*ħω*_*LO* (meV)_	Γ_0_ (eV)
160 nm NWs	0.0885	0.8721	91.3	0.0561
100 nm NWs	0.0916	0.9149	91.3	0.0539
Planar MQW	0.0389	1.2909	91.3	0.0657
